# A Five-Year Review of the Outcomes of Breast Imaging Reporting and Data System 4 Lesions in Hospital Universiti Sains Malaysia

**DOI:** 10.7759/cureus.22757

**Published:** 2022-03-01

**Authors:** Karthikeyan Marthay, Maya Mazuwin Yahya, Tengku Ahmad Damitri Al-Astani Tengku Din, Wan Zainira Wan Zain, Juhara Haron, Michael Pak-Kai Wong, Rosenelifaizur Ramely, Wan Muhammad Mokhzani Wan Mokhter, Siti Rahmah Hashim Isa Merican, Mohd Nizam Mohd Hashim

**Affiliations:** 1 Department of Surgery, School of Medical Sciences, Universiti Sains Malaysia, Kota Bharu, MYS; 2 Breast Cancer Research and Awareness Unit (BestARi), Hospital Universiti Sains Malaysia, Kota Bharu, MYS; 3 Department of Chemical Pathology, School of Medical Sciences, Universiti Sains Malaysia, Kota Bharu, MYS; 4 Breast Cancer Awareness and Research Unit (BestARi), Hospital Universiti Sains Malaysia, Kota Bharu, MYS; 5 Department of Radiology, School of Medical Sciences, Universiti Sains Malaysia, Kota Bharu, MYS

**Keywords:** breast ultrasonography, bi-rads4, biopsy, mammography, breast carcinoma

## Abstract

Purpose: The Breast Imaging Reporting and Data System (BI-RADS) lexicon used in reporting breast imaging has several categories with specific positive predictive values for breast cancer. Among those, BI-RADS 4 is associated with a wider range of risk for breast cancer, which makes the decision for biopsy difficult. The study aim was to determine the malignancy rate and clinical outcomes of BI-RADS 4 lesions in Hospital Universiti Sains Malaysia (HUSM) for a period of five years.

Methods: This was a retrospective study of patients diagnosed by mammographic or ultrasonographic findings with BI-RADS 4 breast lesions in HUSM, Kelantan from July 2015 to June 2020. Data were collected from the medical records and an electronic database. Patients with BI-RADS 4 lesions who underwent biopsy and had a known tissue diagnosis were included in this study. The data was used to calculate the malignancy rate and associated positive predictive factors for breast cancer associated with BI-RADS 4 lesions.

Results: From the mammography and ultrasonography performed at HUSM from July 2015 to June 2020, a total of 256 lesions were categorized as BI-RADS 4. However, only 198 BI-RADS 4 lesions underwent biopsy and were included in the study. Of these 198 lesions, 26.8% were malignant on histopathological examination of the biopsy samples. Simple logistic regression analysis showed that age, diabetes mellitus, hypertension, number of parity, and certain mammogram findings were significantly associated with breast cancer. Invasive breast cancer was the most common type. Fibrocystic disease was the most common benign pathology, followed by fibroadenoma.

Conclusion: The malignancy rate of BI-RADS 4 lesions in HUSM was similar to previously reported rates. A thorough evaluation of positive predictive factors and careful selection of patients for biopsy in BI-RADS 4 lesions will minimize unwanted biopsies and associated patient anxiety, in addition to reducing the health care burden.

## Introduction

Among Malaysian women, breast cancer is the most common cancer and accounts for 31% of all cancers [1]. Mammography and ultrasonography are the most efficient screening tools for early detection of breast cancer and to reduce the morbidity and mortality associated with breast cancer [2].

However, recent advancements in both ultrasonography and mammography and the widespread use of these imaging modalities not only have positive impacts on the management of breast cancer but also have negative effects. The detection of clinically asymptomatic lesions and the need for biopsy to confirm the pathology contribute to psychological distress among affected patients [2].

The Breast Imaging Reporting and Data System (BI-RADS) lexicon developed by the American College of Radiology (ACR) is used to predict the risk of breast malignancy for lesions detected by both mammography and ultrasonography [3] and to standardize the diagnostic features, terminology, and subsequent management recommendations. The decision for biopsy involves using BI-RADS to categorize the breast lesion into low- and high-risk groups. Among the six categories, BI-RADS 4 lesions have the widest range of risk for breast cancer, 2-95% [4]. The uncertain probability of benign and malignant outcomes of BI-RADS 4 lesions challenges clinicians when choosing between surveillance and biopsy as the next step of management. A biopsy is most often performed to confirm a malignancy, although a high number of lesions turn out to be benign.

The high number of false-positive imaging reports in BI-RADS 4 requires careful evaluation of other independent variables that determine the risk of breast cancer. A low-risk BI-RADS 4 lesion can be clinically assessed at regular intervals and followed up rather than subjected to immediate biopsy. Active surveillance instead of invasive clinical intervention of BI-RADS 4 lesions requires judicious selection of patients to achieve an optimum balance between false-positive reports and not missing potential early breast cancer lesions.

The study aim was to identify the clinical and imaging features predictive of malignant BI-RADS 4 breast lesions before invasive intervention. We reviewed and analyzed patients diagnosed with BI-RADS 4 lesions and their respective tissue biopsy results to determine the malignancy rate and associated risk factors that affect the malignancy outcome of BI-RADS 4 lesions in a Malaysian hospital for a period of five years.

## Materials and methods

Study design

This was a five-year retrospective study among patients who underwent mammography and/or ultrasonography breast assessments and was found to have BI-RADS 4 lesions (although the BI-RADS score is categorized into A, B, and C, it is generalized as BI-RADS 4 in our center) at Hospital Universiti Sains Malaysia (HUSM). The data for all patients with BI-RADS 4 lesions from July 2015 to June 2020 were retrieved from the hospital's electronic database and from patient folders collected from the medical records unit.

All participants were identified by their registration number. A proforma developed by researchers included sociodemographic factors, clinical factors (symptoms), indications for mammography, indications for ultrasonography, and biopsy outcomes, and was used to extract variables needed for analysis.

The BI-RADS 4 final assessment category assigned to a particular patient was based on the combined results of both mammography and ultrasonography according to the ACR guidelines. Typically, patients who underwent mammography also had complimentary ultrasonography. However, younger patients with dense breasts only had ultrasonography assessments performed. Data collection included all imaging of patients who underwent two or more repeated imaging examinations during the study period if each imaging was followed by an intervention procedure with available biopsy results.

Various methods have been used to obtain tissue diagnosis, such as fine-needle aspiration cytology, Tru-cut® (Merit Medical, South Jordan, UT) biopsy (which consists of both ultrasound-guided and free-hand), and surgical excision. The cytology or histopathology reports of the participating patients were collected to analyze the malignancy outcomes. In the event of multiple pathologies, the most worrisome pathology was considered in this study. The data were downloaded into Excel and exported to SPSS statistical software (IBM SPSS Statistics for Windows, Version 26.0, IBM Corp., Armonk, NY) for analysis.

Patients who fulfilled the inclusion criteria were those who underwent mammography and/or ultrasonographic breast examination with BI-RADS 4 lesions at HUSM with biopsy. Patients who were diagnosed with BI-RADS 4 lesions but without histopathology or cytology assessment were excluded from the data collection. Patients with incomplete data were also excluded from the study.

## Results

A total of 256 lesions were categorized as BI-RADS 4 from the mammography and/or ultrasonography performed at Hospital USM from July 2015 to June 2020. However, only 198 BI-RADS 4 lesions had biopsies performed and were included in the study for the analysis. Out of the 198 patients, 140 had undergone mammography (with or without ultrasonography). The majority of patients (195) had undergone ultrasonography as a complementary or as a primary investigation due to the younger age group. The most common indication for breast imaging, including mammography and ultrasonography, were palpable breast lumps (60.1%) followed by surveillance for previous cancer diagnosis (16.2%). The most common significant findings in BI-RADS 4 lesions on mammography were negative findings (35.0%) and mass lesions (29.3%). The negative findings referred to BI-RADS 4 lesions were detected by ultrasonography but not detected on mammography and vice versa. The most common finding on ultrasonography was a mass lesion (45.1%). The distribution of the indications and most characteristic findings for both mammography and ultrasonography is summarized in Table 1.

**Table 1:** Clinical indications and imaging characteristics of patients with BI-RADS 4 lesions in HUSM (n = 198) HUSM: Hospital Universiti Sains Malaysia, BI-RADs: Breast Imaging-Reporting and Data System

Imaging characteristics		n (%)
Indications		
Palpable breast lump	119 (60.1)	
Surveillance of previous cancer diagnosis	32(16.2)	
Follow-up specific lesion	14(7.1)	
Nipple discharge	11 (5.6)	
Screening	10 (5.1)	
Axillary lump	6(3.0)	
Breast pain	3(1.5)	
Other	3 (1.5)	
Mammogram findings (n = 140)		
Negative finding (positive findings on ultrasound)	49 (35.0)	
Architectural distortion	8 (5.7)	
Calcifications	29 (20.7)	
Lymph node	7 (5.0)	
Mass lesion	41 (29.3)	
Mass lesion with calcifications	3 (2.1)	
Others (low density lesion, dense breast parenchyma, micro and macrocalcification)	3 (2.1)	
Ultrasound finding, (n = 195)		
Negative findings (positive findings on mammogram)	8 (4.1)	
Calcifications	11 (5.6)	
Ductal abnormality	18 (9.2)	
Lymph node	15 (7.7)	
Mass lesion	88 (45.1)	
Presence of vascularity	47 (24.1)	
Others (cystic lesion, hypoechoic lesion and lobulated lesion)	8 (4.1)	

The malignancy rate of BI-RADS 4 lesions in HUSM during the study period was 26.8%. Out of 198 BI-RADS 4 lesions, 53 were determined to be malignant by tissue biopsy. The proportion per year is shown in Table 2.

**Table 2:** Proportion of malignancies among patients with BI-RADS 4 lesions according to year in HUSM (n = 198) HUSM: Hospital Universiti Sains Malaysia, BI-RADs: Breast Imaging-Reporting and Data System

Year	Malignancy diagnosis, n (%)		Total cases of BI-RADS 4 according to a year
	No (n = 145)	Yes (n = 53)	
(August–December) 2015	9	9	18
(January–December) 2016	12	6	18
(January–December) 2017	6	2	8
(January–December) 2018	38	8	46
(January–December) 2019	49	14	63
(January–July) 2020	31	14	45

Various methods were used for tissue biopsy. The most common technique used was Tru-cut biopsy, which includes both ultrasound-guided and direct Tru-cut biopsy. Out of the 198 lesions, 139 were subjected to Tru-cut biopsy. Ninety-nine lesions were benign, and the remaining 40 lesions were malignant after examination of tissue obtained by Tru-cut. Excision biopsy includes wide local excision with or without hook wire localization. In cases in which the biopsy results were indeterminate by Tru-cut biopsy, an excision biopsy was subsequently performed for confirmation. The biopsy methods and outcomes for the study period are shown in Table 3.

**Table 3:** Comparison of methods of biopsy among patients with BI-RADS 4 lesions in HUSM (n = 198) HUSM: Hospital Universiti Sains Malaysia, BI-RADs: Breast Imaging-Reporting and Data System, FNAC: fine needle aspiration cytology

Biopsy characteristic	Malignancy outcome (n, %)	
	No	Yes
Procedure		
Tru-cut®	99 (68.3)	40 (75.5)
Excision biopsy	42 (29.0)	12 (22.6)
FNAC	2 (1.4)	1 (1.9)
Stereotactic biopsy (utilising Tru-Cut ® needle)	2 (1.4)	0 (0.0)
Total	145(73.2)	53 (26.7)

The BI-RADS 4 lesions were categorized into two groups: the malignant and benign lesion groups. The characteristics of the independent variables of each group are compared and summarized in Table 4. The mean age of the patients was 51.7 (SD, 10.30) years in the malignant group and was 44.4 (SD, 11.48) years in the benign group. The majority of the BI-RADS 4 lesion patients were Malay, followed by Chinese. The age of menopause was also analyzed in both groups; the majority of the patients (132) were premenopausal. In general, most of the patients had no family history of malignancy or used hormone replacement therapy.

**Table 4:** Comparison of malignancies among patients with BI-RADS 4 lesions in HUSM by patient characteristics (n = 198) HUSM: Hospital Universiti Sains Malaysia, BI-RADs: Breast Imaging-Reporting and Data System, SD: standard deviation

Patient characteristics	Malignancy diagnosis	
	No (n = 145)	Yes (n = 53)
Mean age (SD)	44.48 (11.48)	51.72 (10.30)
Ethnicity		
Malay	132	46
Chinese	13	7
Comorbidities (n = 179; missing data 19)		
Yes	43	21
Diabetes mellitus type 2	15	14
Hypertension	28	17
Hyperlipidemia	13	8
Chronic kidney disease	1	0
Ischemic heart disease	0	0
Other	9	1
No	91	24
Parity (n = 179; missing data 19)	Mean 2.68 (SD1.99)	Mean 3.91 (SD 2.09)
History of pregnancy, (n = 179 ; missing data 19)		
No/nulliparous	31	5
Yes	102	41
Age of Menarche (n = 171; missing data 27)	Mean 13.05 (SD 1.30)	Mean 13.14 (SD1.41)
Age of menopause (n = 47)	Mean 50.48 (SD 4.95)	Mean 50.44 (SD2.77)
Hormone replacement therapy intake (n = 178; missing data 20)		
No	101	28
Yes	33	16
Family history (n = 178; missing data 20)		
No	105	32
Yes	30	11

Most patients in this study group had no known medical illnesses. However, among the patients with medical illnesses, diabetes mellitus was the most common disease in both the malignant and benign groups. The other comorbidities included diseases such as bronchial asthma, thalassemia, and congenital heart disease, which only a few patients had. A comparison of imaging findings among the patients with BI-RADS 4 lesions in HUSM by clinical indication and imaging characteristics is shown in Table 5.

**Table 5:** Comparison of imaging findings among patients with BI-RADS 4 lesions in HUSM by clinical indications and imaging characteristics, August 2015 to July 2020 (n = 198) HUSM: Hospital Universiti Sains Malaysia, BI-RADs: Breast Imaging-Reporting and Data System

Imaging characteristic	Malignancy outcome: n (%)	
	No, n = 145	Yes, n = 53
Indications		
Palpable breast lump	79 (54.9)	40 (75.5)
Axillary lump	5 (3.4)	1 (1.9)
Breast pain	3 (2.1)	0 (0.0)
Nipple discharge	7 (4.8)	4 (7.5)
Screening	10 (6.9)	0 (0.0)
Surveillance	26 (17.9)	6 (11.3)
Follow-up specific lesion	12 (8.3)	2 (3.8)
Other	3 (2.1)	0 (0.0)
Mammogram finding, (n = 140)		
Negative finding (when BI-RADS 4 lesions detected on ultrasound)	45 (45.5)	4 (9.8)
Architectural distortion	2 (2.0)	6 (14.6)
Calcifications	22 (22.2)	7 (17.1)
Lymph node	4 (4.0)	3 (7.3)
Mass lesion	23 (23.2)	18 (43.9)
Mass lesion with calcifications	2 (2.0)	1 (2.4)
Others	1 (1.0)	2 (4.9)
Ultrasound finding, (n = 195)		
Negative findings	6 (4.2)	2 (3.8)
Calcifications	7 (4.9)	4 (7.7)
Ductal abnormality	17 (11.9)	1 (1.9)
Lymph node	9 (6.3)	6 (11.5)
Mass lesion	68 (47.6)	20 (38.5)
Presence of vascularity	33 (23.1)	14 (26.9)
Others	3 (2.1)	5 (9.6)

Based on Table 6, there were four clinical factors and one imaging factor (Table 7) that had significant associations with malignancies, which were age, diabetes mellitus, hypertension, number of parity, and mammography findings. Details of the simple logistic regression analysis for clinical factors and imaging factors are summarized in Tables 6-7.

**Table 6:** Simple logistic regression for clinical factors associated with malignancy among patients with BI-RADS 4 lesions in HUSM, August 2015 to July 2020 HUSM: Hospital Universiti Sains Malaysia, BI-RADs: Breast Imaging-Reporting and Data System, OR: odds ratio, CI: confidence interval

Variables	Malignant				
	No, n = 145	Yes, n = 53	Crude β	Crude OR (95% CI)	p-value
Age	145	53	0.06	1.06 (1.03, 1.10)	<0.001
Ethnicity					
Malay	132	46	0	1	
Chinese	13	7	0.44	1.55 (0.55, 4.02)	0.383
Comorbid (n = 179 ; missing data 19)					
No	91	24	0	1	
Yes	43	21	0.62	1.85 (0.93, 3.70)	0.080
Diabetes mellitus status					
No	119	31	0	1	
Yes	15	14	1.28	3.58 (1.56, 8.26)	0.003
Ischemic heart disease status					
No	134	45	0	1	
Yes	0	0	-	-	-
Hypertension status					
No	106	28	0	1	
Yes	28	17	0.83	2.30 (1.10, 4.78)	0.026
Chronic kidney disease status					
No	133	45	0	1	
Yes	1	0	-	-	-
Dyslipidemia status					
No	121	37	0	1	
Yes	13	8	0.70	2.01 (0.75, 5.16)	0.151
Parity	133	46	0.30	1.35 (1.14, 1.63)	0.001
Menarche age (years)	128	43	0.05	1.05 (0.81, 1.37)	0.690
Menopause age (years)	29	18	0.00	1.00 (0.86, 1.16)	0.976
On hormone replacement therapy					
No	101	28	0	1	
Yes	33	16	0.56	1.75 (0.83, 3.61)	0.133
Positive family history (n = 178; missing data 20)					
No	105	32	0	1	
Yes	30	11	0.18	1.20 (0.53, 2.62)	0.649

**Table 7:** Simple logistic regression for imaging factors associated with malignancy among patients with BI-RADS 4 lesions in HUSM (n = 198) HUSM: Hospital Universiti Sains Malaysia, BI-RADs: Breast Imaging-Reporting and Data System, FNAC: Fine Needle Aspiration Cytology, OR: odds ratio, CI: confidence interval

Variable	Malignant, n				
	No, n = 145	Yes, n = 53	Crude β	Crude OR (95% CI)	p-value
Indication					
Palpable breast lump	79	40	0	1	
Axillary lump	5	1	−0.93	0.40 (0.02, 2.56)	0.404
Breast pain	3	0	-	-	-
Nipple discharge	7	4	0.12	1.13 (0.28, 3.97)	0.854
Screening	10	0	-	-	-
Surveillance	26	6	−0.79	0.46 (0.16, 1.13)	0.111
Follow-up specific lesion	12	2	−1.11	0.33 (0.05, 1.28)	0.159
Other	2	0	-	-	-
Mammogram finding					
Negative finding	45	4	0	1	
Architectural distortion	2	6	3.52	33.75 (5.81, 294.73)	<0.001
Calcifications	22	7	1.28	3.58 (0.98, 14.91)	0.060
Lymph node	4	3	2.13	8.44 (1.31, 54.86)	0.021
Mass lesion	23	18	2.18	8.80 (2.90, 33.27)	<0.001
Mass lesion with calcifications	2	1	1.73	5.62 (0.23, 73.67)	0.194
Others	1	2	3.11	22.50 (1.80, 554.57)	0.019
Ultrasound finding					
Negative findings	6	2	0	1	
Calcifications	7	4	0.54	1.71 (0.24, 15.75)	0.601
Ductal abnormality	17	1	−1.73	0.18 (0.01, 2.16)	0.187
Lymph node	9	6	0.69	2.00 (0.32, 16.94)	0.476
Mass lesion	68	20	−0.13	0.88 (0.19, 6.34)	0.884
Presence of vascularity	33	14	0.24	1.27 (0.26, 9.41)	0.783
Others	3	5	1.61	5.00 (0.65, 53.70)	0.142
Biopsy procedure					
Excision biopsy	42	12	0	1	
FNAC	2	1	0.56	1.75 (0.08, 19.85)	0.659
Stereotactic biopsy	2	0	-	-	-
Tru-cut®	99	40	0.35	1.41 (0.69, 3.06)	0.358

For multivariable analysis with multiple logistic regressions, significant variables associated with malignancy were identified from the simple logistic regression. Multiple logistic regressions showed that only mammographic findings, which included mass lesions and architectural distortions, were associated with malignancies among patients with BI-RADS 4 lesions. The details of the multiple logistic regressions are summarized in Table 8. The most common diagnoses were fibrocystic disease (24.8%) and fibroadenoma (19.3%) for non-malignant BI-RADS 4 patients and invasive breast carcinoma (75.5%) for malignant patients. The outcomes of the patients are summarized in Tables 9-10.

**Table 8:** Multiple logistic regression for factors associated with malignancy among patients with BI-RADS 4 lesions in HUSM (n = 198) HUSM: Hospital Universiti Sains Malaysia, BI-RADs: Breast Imaging-Reporting and Data System, OR: odds ratio, CI: confidence interval

Variable	Crude β	Crude OR (95% CI)	p-value	Adjusted β	Adjusted OR (95% CI)	p-value
Age	0.06	1.06 (1.03, 1.10)	<0.001	0.06	1.06 (0.99, 1.13)	0.103
Diabetes mellitus status						
No (reference)	0	1		0	1	
Yes	1.28	3.58 (1.56, 8.26)	0.003	0.47	1.60 (0.34, 7.82)	0.554
Hypertension status						
No (reference)	0	1		0		
Yes	0.83	2.30 (1.10, 4.78)	0.026	−0.11	0.90 (0.22, 3.24)	0.871
Parity	0.30	1.35 (1.14, 1.63)	0.001	0.19	1.20 (0.94, 1.56)	0.141
Mammogram finding						
Negative finding (Reference)	0	1		0		
Architectural distortion	3.52	33.75 (5.81, 294.73)	<0.001	3.33	28.00 (4.43, 256.98)	0.001
Calcifications	1.28	3.58 (0.98, 14.91)	0.060	1.08	2.94 (0.63, 14.55)	0.168
Lymph node	2.13	8.44 (1.31, 54.86)	0.021	1.50	4.48 (0.39, 45.63)	0.204
Mass lesion	2.18	8.80 (2.90, 33.27)	<0.001	2.00	7.36 (2.27, 29.25)	0.002
Mass lesion with calcifications	1.73	5.62 (0.23, 73.67)	0.194	-	-	-
Others	3.11	22.50 (1.80, 554.57)	0.019	2.06	7.84 (0.24, 248.55)	0.194

**Table 9:** Clinical outcome of non-malignant patients with BI-RADS 4 lesions in HUSM (n = 145) HUSM: Hospital Universiti Sains Malaysia, BI-RADs: Breast Imaging-Reporting and Data System. Others include: granulomatous mastitis, mastitis, lactational changes, accessory breast, fat necrosis, sclerosing adenosis, lymphadenitis, fibrous mastopathy, nodular pseudoangiomatous stromal hyperplasia, myofibroblastoma, reactive lymphadenopathy, complex sclerosing lesion, periductal mastitis, chronic inflammation, fibromuscular tissue.

Histopathology outcome	n (%)
Fibrocystic changes	36 (24.8)
Fibroadenoma	28 (19.3)
Others	20 (13.8)
Inflammatory	13 (9.0)
Benign proliferative lesion	12 (8.3)
Papilloma	11 (7.6)
Adenosis	8 (5.5)
Lactating adenoma	4 (2.8)
Fibrosis	3 (2.1)
Phylloides	3 (2.1)
Benign fibroepithelial lesion	2 (1.4)
Ductal hyperplasia	2 (1.4)
Ductal ectasia	2 (1.4)

**Table 10:** Clinical outcome of malignant patients with BI-RADS 4 lesions in HUSM (n = 53) HUSM: Hospital Universiti Sains Malaysia, BI-RADs: Breast Imaging-Reporting and Data System

Histopathology outcome	n (%)
Invasive ductal carcinoma	40 (75.5)
Ductal carcinoma in situ	5 (9.4)
Papillary breast carcinoma	2 (3.8)
Invasive lobular carcinoma	2 (3.8)
Metastatic breast carcinoma	1 (1.9)
Mucinous carcinoma	1 (1.9)
Tubular carcinoma	1 (1.9)

## Discussion

Both mammography and ultrasonography continue to be the recommended screening tools for diagnosing early-stage breast cancer [5]. Although both imaging modalities are able to detect clinically occult malignancies, their potential disadvantages need to be considered. The risk of over-diagnosis, negative tissue biopsy, and associated anxiety, as well as radiation exposure from mammography, are the possible negative effects on patients being investigated for breast lesions [2]. BI-RADS were developed by ACR for systematic and uniform reporting of breast imaging results [3].

The BI-RADS lexicon consists of several final assessment categories, which predict the risk of developing breast cancer of a lesion detected by mammography or ultrasonography. Among the BI-RADS final assessment categories, BI-RADS 4 describes suspicious lesions and is associated with a wider range of risks for malignancy [6].

This retrospective analysis of BI-RADS 4 lesions for five years in HUSM showed that the malignancy rate was 26.8%. This proportion of malignancies is comparable to the proportion of BI-RADS 4 lesions in Bangkok, Thailand, with a positive predictive value of 21% [7]. Our study in HUSM reflects the false-positive breast imaging and the number of negative biopsies performed for benign breast lesions. Breast Cancer Surveillance Consortium data in the United States, which consists of data from 1.6 million women, indicates that 66.8% of biopsy results were non-malignant [2]. Moreover, the cumulative risk of false-positive imaging in women undergoing yearly mammograms has previously been calculated to be 49.1% to 61.3% over 10 years [2]. Imaging examinations performed for non-malignant lesions are associated with anxiety and emotional disturbance in patients, in addition to increased health care expenditures.

Many factors are involved in deciding to perform a biopsy on patients presenting with breast lesions. Although it is undeniable that the positive predictive values of BI-RADS guide most clinicians, there are other variables, such as age, the parity index, and family history of breast cancer, that determine the risk of breast cancer in a patient. Using logistic regression models, we demonstrated the role of other independent variables, such as age, diabetes mellitus, hypertension, number of parities, and mammogram findings, that affect the final diagnosis of BI-RADS 4 lesions in HUSM.

Patient age was an independent variable that needs to be considered in deciding to perform biopsies of BI-RADS 4 lesions. The descriptive analysis of this study showed that the mean age was higher in the malignancy group than in the benign group (mean age, 51.7 years vs. 44.4 years). Our analysis showed that with increasing age, the odds ratio of 1.06 was significantly higher (p<0.001) for breast cancer in association with a BI-RADS 4 lesion. These odds ratios are comparable to those of the study by Wiratkapun et al. in 2010, which showed that increasing patient age was associated with an increasing probability of the lesions being malignant (odds ratio of 1.02, p<0.001) [7].

Diabetes mellitus and hypertension are associated with a significant risk of malignancy in BI-RADS 4 lesions. The simple logistic regression analysis of this study showed that diabetic patients with BI-RADS 4 lesions had a three to four times higher risk of developing breast cancer than their healthier counterparts, and the risk of malignancy was increased 2.3-fold in hypertensive patients. Diabetes mellitus is characterized by insulin resistance and a hyperinsulinemic state. Insulin has mitogenic effects on breast tissue, and insulin receptors are frequently over-expressed in breast cancer cells [8]. A systematic review and meta-analysis by Han et al. in 2017 showed that women with hypertension may have a 15% increased risk of breast cancer [9].

There were various indications for mammography and ultrasonography in HUSM during the study period. Most patients in this study presented with palpable breast lumps (60.1%) prior to breast imaging. Almost 16% of the study population had breast imaging for surveillance. The descriptive analysis shows that 40 out of 53 patients with malignant BI-RADS 4 lesions based on tissue diagnosis had palpable breast lumps as the initial presentation. This finding is supported by data from Thailand showing that compared with other indications for breast imaging, a palpable mass had an odds ratio of 2.13 for breast cancer [7].

A retrospective review of BI-RADS 4 imaging showed that the most common characteristic finding on ultrasonography in this study was a mass lesion. On the other hand, a negative finding followed by mass lesions is frequently encountered in mammography. A negative finding refers to a BI-RADS 4 lesion identified on breast ultrasonography but no significant lesion detected on mammography. As shown by multiple logistic regression analysis, architectural distortion and mass lesions on mammography are significant risk factors for breast cancer in patients with BI-RADS 4 lesions. In contrast to mammography, although a mass lesion is the most common characteristic finding in ultrasonography, there is no significant odds ratio associated with breast cancer.

Studies have demonstrated that not all cancer lesions are diagnosed by mammography and that breast density affects the outcome of mammographic final interpretations. Results from a Dutch screening agency's digital mammography assessment from 2003 to 2011 [10] showed that the sensitivity of mammography was 85.7% for women with fatty breasts. The sensitivity was greatly reduced to 61% for women with extremely dense breast tissue. These observations suggest that the role of mammography in high-density breast tissue is suboptimal. On the other hand, ultrasonography is patient-friendly and has no harmful effects of ionizing radiation regardless of breast density. The literature indicates that, compared with mammography used alone, the breast cancer detection rate increases when mammography and ultrasonography are combined [11,12]. However, an increased number of false-positive interpretations and benign biopsy results are potential unwanted effects of a combined imaging modality.

The BI-RADS 4 lesions in this study were predominantly benign. Almost 75% of malignant lesions were invasive ductal carcinomas. The second most common pathology in the malignant group was ductal carcinoma in situ (DCIS). The benign pathologies covered a spectrum of diseases, mainly fibrocystic changes, fibroadenoma, inflammatory lesions, and papilloma. These pathological findings are consistent with those of other similar studies [7,13]. DCIS contributed to almost 10% of the malignant lesions in this study. This finding is consistent with data from the United States in which DCIS accounts for 12-15% of newly diagnosed breast cancer each year in that region [14]. The mammogram screening program resulted in an increased number of DCIS detections in otherwise asymptomatic women. Although the dominant feature of DCIS is suspicious microcalcifications, 10-20% of DCIS in patients is present with no calcification on mammography [14].

The Tru-cut biopsy was the most popular technique used for biopsy in this study and is preferred over other biopsy techniques because it can be performed in an outpatient setting without the need for an operating theater and general anesthesia. Compared with fine-needle aspiration cytology, a Tru-cut biopsy enables a definitive diagnosis by providing core tissue for histopathological examination [15]. The Tru-cut biopsy can differentiate between atypical hyperplasia and malignancies and guide surgeons in planning treatment strategies. Stereotactic biopsy is used for lesions that cannot be localized by ultrasonography for tissue diagnosis, especially in microcalcifications of BI-RADS 4 lesions. Stereotactic biopsy performed with a large-core vacuum-assisted instrument has a sensitivity equal to that of hook-wire localization and excision. Moreover, stereotactic biopsy is safer and more cost-effective than excision biopsy [16].

In contrast to the other findings in the current literature, parity in this study was found to be a risk factor for breast cancer in patients with BI-RADS 4 lesions. The analysis indicated that higher parity was associated with the malignant group of BI-RADS 4 lesions. The simple logistic regression analysis showed that parity had an odds ratio of 1.35 for breast cancer, which was significant. This association is possible due to the effects of multiple confounding variables that were not analyzed in this study. More detailed studies are required to confirm the relationship between parity and breast cancer in patients with BI-RADS 4 lesions, as this study is retrospective in nature and has its weaknesses.

## Conclusions

The study results showed that 26.8% of the patients with BI-RADS 4 lesions had biopsy-proven malignancies. Although this finding is consistent with current evidence, the decision for biopsy in BI-RADS 4 lesions should be individualized. The study showed that mammographic findings, such as mass lesions and architectural distortions, are significant risk factors for malignancy in BI-RADS 4 lesions. These study findings need to be confirmed by doing a prospective study of the BI-RADS 4 lesions in other centers in Malaysia to increase the breast cancer detection rate. Complete BI-RADS A, B, and C classifications should be done in order to further classify the BI-RADS 4 and the association to malignancy accurately.

## References

[1] Tan MM, Ho WK, Yoon SY (2018). A case-control study of breast cancer risk factors in 7,663 women in Malaysia. PLoS One.

[2] Hsu W, Zhou X, Petruse A (2019). Role of clinical and imaging risk factors in predicting breast cancer diagnosis among BI-RADS 4 cases. Clin Breast Cancer.

[3] Obenauer S, Hermann KP, Grabbe E (2005). Applications and literature review of the BI-RADS classification. Eur Radiol.

[4] Lei CQ, Wei W, Liu ZY, Xiong QQ (2019). Radiomics analysis for pathological classification prediction in BI-RADS category 4 mammographic calcifications. J Clin Oncol.

[5] Suttawas A (2018). Positive predictive value and biopsy rate of breast cancer in BI-RADS category 4 and 5 breast lesions. Reg 4-5 Med J.

[6] Torres-Tabanera M, Cárdenas-Rebollo JM, Villar-Castaño P, Sánchez-Gómez SM, Cobo-Soler J, Montoro-Martos EE, Sainz-Miranda M: (2012). Analysis of the positive predictive value of the subcategories of BI-RADS® 4 lesions: Preliminary results in 880 lesions. Radiología (English Edition).

[7] Wiratkapun C, Bunyapaiboonsri W, Wibulpolprasert B, Lertsithichai P (2010). Biopsy rate and positive predictive value for breast cancer in BI-RADS category 4 breast lesions. J Med Assoc Thai.

[8] Larsson SC, Mantzoros CS, Wolk A (2007). Diabetes mellitus and risk of breast cancer: a meta-analysis. Int J Cancer.

[9] Han H, Guo W, Shi W, Yu Y, Zhang Y, Ye X, He J (2017). Hypertension and breast cancer risk: a systematic review and meta-analysis. Sci Rep.

[10] Wanders JO, Holland K, Veldhuis WB (2017). Volumetric breast density affects performance of digital screening mammography. Breast Cancer Res Treat.

[11] Sprague BL, Stout NK, Schechter C (2015). Benefits, harms, and cost-effectiveness of supplemental ultrasonography screening for women with dense breasts. Ann Intern Med.

[12] Melnikow J, Fenton JJ, Whitlock EP, Miglioretti DL, Weyrich MS, Thompson JH, Shah K (2016). Supplemental screening for breast cancer in women with dense breasts: a systematic review for the US Preventive Services Task Force. Ann Intern Med.

[13] Chaitanya INVL, Prabhala S, Annapurna Srirambhatla, Deshpande AK (2020). Comparison of histopathologic findings with BIRADS score in Tru-cut biopsies of breast lesions. IJPRP.

[14] Su X, Lin Q, Cui C, Xu W, Wei Z, Fei J, Li L (2017). Non-calcified ductal carcinoma in situ of the breast: comparison of diagnostic accuracy of digital breast tomosynthesis, digital mammography, and ultrasonography. Breast Cancer.

[15] Samantaray S, Panda N, Besra K (2017). Utility of Tru-Cut biopsy of breast lesions-an experience in a regional cancer center of a developing country. J Clin Diagn Res.

[16] Tsai HY, Chao MF, Ou-Yang F, Kan JY, Hsu JS, Hou MF, Chiu HC (2019). Accuracy and outcomes of stereotactic vacuum-assisted breast biopsy for diagnosis and management of nonpalpable breast lesions. Kaohsiung J Med Sci.

